# Aneurism of the Popliteal Space Treated Successfully by Compression

**Published:** 1850-07

**Authors:** 


*Aneurism of the Popliteal Space treated successfully by Compresssson.*—Mr. Banon communicated to the Surgical Society of Ireland (Nov. 17th, 1849) a very interesting case of this. The subject of it was a laborer, 27 years of age, admitted into Jervis Street Hospital, Sept. 11th, 1849. He stated that, fifteen weeks previously, he had suffered from a stiffness of the left knee, accompanied by shooting pains towards the ankle, which he attributed to a strain while lifting a heavy weight. Three or four days subsequently, he first observed a tumor about the size of a walnut in the ham of the same limb. In a fortnight, the swelling had attained the size of an apple. The leg and foot became somewhat swollen, and the stiffness in the knee had increased, with a sense of numbness. An unusual throbbing in the part at this time also attracted his attention. He acknowledged that he had drunk freely for several years, but stated that his general health had been invariably good. Upon a careful examination of the chest, no disease of the lungs or heart could be detected, and, indeed, all his vital organs appeared in a healthy state. He was induced to apply for medical relief by the alarm which the rapid increase of the aneurism had latterly occasioned him. Upon examination, a tumour, fully the size of a very large orange, was seen pulsating in and occupying the entire of the left popliteal space. It was soft and yielding over its whole surface, and capable of being considerably diminished by pressure. A very loud bruit de soufflet was audible on applying the stethoscope. Pressure on the femoral artery caused a considerable diminution in size and a cessation of all pulsation in the tumour. The leg and foot were somewhat edematous, and he complained much of pain about the knee and ankle.

Sept. 14th. He was bled to ten ounces, and ordered a drachm of compound powder of jalap, and directed to be put on low diet.

16th. At eleven o'clock this morning, I commenced compression of the femoral artery by the application of two clamps—one adjusted to pass round the pelvis, and to make pressure immediately below Poupart's ligament; the other (Mr. Carte's) was brought to bear on the vessel about the middle of the thigh. The usual mode of relaxing one instrument after having screwed down the other, was followed, and carefully attended to by our resident pupil, Mr. Fitzgibbon, and by this means a certain amount of pressure was constantly kept up on the artery until eleven o'clock at night, but at no time to such an extent as to stop all pulsation in the tumour. After having thus borne the pressure for twelve hours, he became restless and uneasy, and the swelling of the leg having increased, accompanied by venous congestion, the instruments were both relaxed, and only again screwed down at intervals during the night.

17th. He had no sleep during the last night, although a full anodyne had been administered. The pulsation is somewhat less, and the aneurism no longer yields to the slightest pressure as before, having become decidedly firmer.

On examination of the limb, the edema is still present. The articular arteries are felt pulsating on each side of the joint. Pressure was again applied and continued by the alternate action of the clamps.

18th. Edema increased; patient complained more of the numbness and shooting pains. He was, however, anxious that the compression of the artery should be continued, on relaxing which for a moment, for the first time since yesterday morning, a greater degree of firmness of the tumour is observed, and a much fainter pulsation. The bruit de soufflet, before so loud, is now scarcely audible.

20th. The pressure has been kept up without intermission, since last report, day and night, the only unpleasant effects being loss of rest and a burning pain in the knee, with an increase of the edematous swelling. The temperature of the leg and foot was at times somewhat lower than that of the opposite limb. Warm jars were applied, from which he derived much comfort. Our patient had now been three days and nights without any sleep whatever. I therefore directed that the instrument should be loosened, on doing which scarcely any pulsation returned to the aneurism. The pressure had been scarcely removed from the limb when he fell into a profound sleep, of which our resident pupil adroitly took advantage by screwing down Carte's instrument rather tighly on the artery, without, however, awaking him for six hours, when we had the pleasure to find that all pulsation had ceased in the tumour, nor did it ever subsequently return.

A slight pressure was kept up for a day or two longer, when the instruments were finally taken off. The edematous swelling of the limb disappeared rapidly. The swelling in the popliteal space, up to the time of his leaving the hospital on the 1st of October, continued to diminish in size, and became harder, and every day he was regaining the use of the limb. I have since watched this man's progress, and found it to be most satisfactory. The tumour has become gradually smaller, and is now of so little inconvenience that he is able to work at the custom-house docks, where he is obliged frequenty to carry very heavy loads. A cast, taken a month subsequent to his leaving the hospital, which I now exhibit, shows that the original size of the tumour had diminished at least one-half. Through some inadvertence, a cast was not taken previous to the application of compression.

I should here mention that the pulsation of the femoral artery could be distinctly felt, after the instruments had been finally removed from the limb, over its whole course until it approached the popliteal space; thus proving that no such thing as obliteration of the vessel at the seat of pressure takes place, the process of cure occasioning it only in the situation of the aneurism, where it converts the artery into an impervious ligamentous cord.

Mr. B., in his remarks on this case, observed that "the fact of the whole tumour having become solid in less than four days after its first application, is a circumstance which should be noted by those who consider the proceeding both irksome and tedious, and consequently inferior to the ligature, and should, in my opinion, go far to convert the most skeptical. It will be always necessary to observe caution as to the use of too great a degree of pressure, as abrasion, or even sloughing, may be the consequence. The edema, the purplish hue of the limb, the falling of the temperature, all present in this case, should be watched with the greatest anxiety. I considered myself justified in continuing the pressure under these circumstances, from having been able to feel the pulsation in the articular arteries and anterior tibial, the former of which were apparently increased in size; thus showing the activity of the collateral circulation going on in the limb; but, had the decrease in temperature become more marked, I should, of course, have relaxed the clamps."

*Dublin Medical Press*, Nov. 28th, 1849.

In the same Journal for February, we find a report of a case of popliteal aneurism treated by compression. It was treated on Dr. Billingham's plan. The case is exceedingly interesting, although a fatal issue was the result. This fatality, however, was in consequence of the bursting of an abdominal aneurism, and the following conclusions seem justified by the facts:

"That no plan of treatment could have saved the patient ultimately from death by the cause stated.

"That the treatment of popliteal aneurisn in this case, by pressure in groin, or three inches below it, did not morbidly affect the part of the artery pressed on, which remained pervious, and exhibited no change of structure whatever.

"The healthy and unaffected condition of the artery, at the point of pressure, refutes the statement of those pathologists who state that pressure injures the vessel in case it should be subsequently necessary to operate on it.

"That the aneurism in ham was in that process of cure by the gradual deposition of layers of fibrine proceeding from surface to center of sac.

"That the inch and a half of vessel rendered impervious immediately above the aneurism, contained a pedicle or cone of same substance (fibrine,) which occasioned the obstruction of its contents."

